# *Garcinia cambogia* Extract Increased Hepatic Levels of Lipolysis-Stimulated Lipoprotein Receptor and Lipids in Mice on Normal Diet

**DOI:** 10.3390/ijms242216298

**Published:** 2023-11-14

**Authors:** Marine Hanse, Samina Akbar, Hamed Layeghkhavidaki, Frances T. Yen

**Affiliations:** 1 EA 4422 Lipidomix Laboratory, University of Lorraine, 54505 Nancy, France; 2 Quality of Diet and Aging Team, UR 3998 Animal and Functionality of Animal Products Laboratory, University of Lorraine, 54505 Nancy, France

**Keywords:** *Garcinia cambogia*, lipid homeostasis, lipoprotein receptors, liver, triglycerides, mice

## Abstract

*Garcinia cambogia* extract (GCE) is a popular weight-loss supplement that also lowers plasma triglyceride (TG) levels. We hypothesized that GCE-mediated inhibition of ATP citrate lyase and thereby hepatic TG production could lead to compensatory mechanisms, including increased hepatic TG uptake via lipoprotein receptors. GCE (20 mg/day) administered 40 days orally to female C57BL/6Rj mice on a standard chow diet led to a decrease in both plasma fasting and post-prandial TG-rich lipoprotein levels, but with no significant change in body weight gain. Lipolysis stimulated lipoprotein receptor (LSR) protein levels, but not those of LDL-receptor, were increased as compared to controls. Mouse Hepa1-6 cells treated with the GCE active ingredient, hydroxycitrate, also led to increased LSR protein levels. Hepatic total cholesterol, TG, and muscle TG contents were higher in GCE-treated animals as compared to controls, whereas adipose TG levels were unchanged. LSR and LDL-receptor protein levels were correlated with liver total cholesterol, but only LDL-receptor was associated with liver TG. These results show that GCE treatment in mice on a standard chow diet led to significantly increased liver and muscle lipids, with no significant change in adipose tissue TG levels, which should be considered in the long-term use of GCE.

## 1. Introduction

Obesity is a significant risk factor for cardiovascular and metabolic diseases, and its increasing incidence has become a major health concern in industrialized countries. Because of this, reducing excess fat mass has been the target of numerous strategies and treatments, including the use of plant extracts as weight-loss supplements with varying rates of success [1,2]. The extract of the fruit rind of *Garcinia cambogia* (GC), a medicinal plant that grows in Africa and Southeast Asia, is a widely used treatment [3]. Oral intake of the GC extract (GCE) leads to weight loss [4] by decreasing food intake and lowering plasma triglyceride (TG) levels in human subjects as well as in animal models [5,6]. These effects have been attributed to the decreased lipogenesis due to the GCE active ingredient, hydroxycitrate (HCA), which is a strong competitive inhibitor of the liver enzyme ATP citrate lyase (ACL) [7,8,9,10]. ACL catalyzes the production of acetyl-CoA from citrate and is used for de novo fatty acid synthesis and TG production [11]. HCA inhibition of ACL therefore decreases TG output as VLDL from the liver [12], contributing to decreased plasma TG and decreased weight gain.

However, lipid homeostasis is tightly regulated in the liver, and the inhibition of hepatic de novo lipogenesis could lead to compensatory mechanisms such as increasing TG influx into the liver to maintain TG balance. Such is the case for regulation of cholesterol levels via the SREBP pathway [13], which acts as a cholesterol sensor. For example, statin inhibition of HMG CoA reductase and de novo cholesterol synthesis lowers hepatic cholesterol levels, which activates the SREBP pathway to increase transcription of genes involved not only in cholesterol synthesis but also those involved in cholesterol uptake, such as the LDL-receptor (LDL-R) [14,15]. Increased hepatic uptake of LDL via the LDL-R thus contributes towards maintaining cholesterol balance. Similar mechanisms may exist for the regulation of TG levels, and GCE’s mechanism of action provides the means to determine if inhibition of hepatic de novo lipogenesis can also modify hepatic uptake of TG.

The removal of TG from the circulation is mediated via lipoprotein receptors that bind the apoE component of TG-rich lipoproteins (TGRL) [16]. The LDL-R can bind both apoB and apoE, but normal clearance of TGRL in LDL-R-deficient patients or animal models indicated the presence of other LDL-R-independent pathways [17,18]. For the LDL-R-related protein (LRP1), which is an LDL-R family member, accumulation of cholesterol but not TG is observed in hepatic LRP1 KO mice lacking the LDL-R [19]. The lipolysis-stimulated lipoprotein receptor (LSR) is an apoB,E receptor that binds with the highest affinity to TGRL and has been shown to contribute to the removal and endocytosis of these particles during the post-prandial phase [20,21]. Furthermore, LSR expression has been linked to transcriptional factors involved in fatty acid uptake, trafficking, and oxidation, as well as TG storage and lipolysis [22]. We recently reported that the epistatic interaction between LSR and APOE gene variants influences blood lipid profiles in a healthy cohort [23]. LSR may thus be important in the regulation of liver lipid homeostasis, particularly in processes involving the regulation of hepatic TG balance. 

Here, we hypothesize that GCE-mediated inhibition of hepatic TG production leads to increased hepatic removal of TG-rich lipoproteins from the circulation, potentially leading to hepatic lipid accumulation. To test this, we investigated the effect of GCE treatment in mice on protein levels of the hepatic lipoprotein receptors, LSR and LDL-R, and liver lipid composition.

## 2. Results

### 2.1. In Vivo Study of GCE Treatment in Mice

#### 2.1.1. Body Mass Gain Measurement

**Figure 1:**
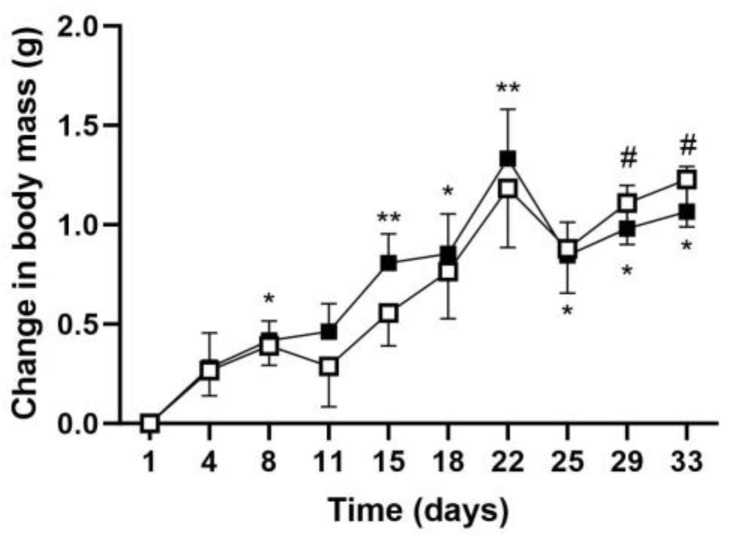
Effect of GCE on body mass in C57BL/6J mice. Ten-week-old female C57BL/6J mice received 300 µL of 50% (*v*/*v*) corn oil emulsion in physiological saline containing 20 mg GCE (■, GCE, *n* = 10); control mice received only the corn oil emulsion (CTRL, ☐, *n* = 10), as described in Materials and Methods. Body mass was monitored at the indicated times; changes in body mass compared to Day 1 are shown as mean ± SEM. Statistical analyses were performed using a 2-way ANOVA (# *p* < 0.05 as compared to day 1 CTRL; * *p* < 0.05 or ** *p* < 0.01, as compared to Day 1 GCE).

Wild-type C57BL/6J female mice from the same lot were separated into two groups. One group received a gavage of 300 µL of a 50% (*v*/*v*) corn oil emulsion in physiological saline containing 20 mg of GCE (*n* = 10, GCE); the control group (*n* = 10, CTRL) received vehicle alone. Both groups were maintained on the same standard chow diet *ad libitum* and monitored for body weight gain (every 4 days) and food intake (weekly). At the start of the experiment, body masses were not significantly different: 18.8 ± 0.22 g (CTRL) and 18.9 ± 0.13 (GCE). There were small but significant increases in body mass in each group as compared to Day 1 of the same group (Figure 1). However, no significant changes in weight gain were observed between the two groups.

Food intake was monitored weekly and used to calculate the total amount of kcal consumed, including that administered via gavage (Table 1), assuming a caloric density of 3.1 kcal/g of the standard chow diet, of which 18% is fat-based, and a caloric density of 8.68 kcal/g for corn oil with a density of 0.91 g fat/mL. 

Dietary intake was significantly lower (7% decrease, *p* < 0.01) in GCE as compared to CTRL, but only after the first week (Day 8). After this, total daily caloric intake was not significantly different between the CTRL and GCE groups for the remaining period. There was a small but significantly lower food intake in the CTRL group on Day 29 as compared to Day 8. The gavage of corn oil increased % kcal as fat from 18% in the standard chow diet to between 23 and 24%. Both groups received the same volume of corn oil, and the statistical differences for fat intake were the same as those observed for dietary intake.

**Figure 2:**
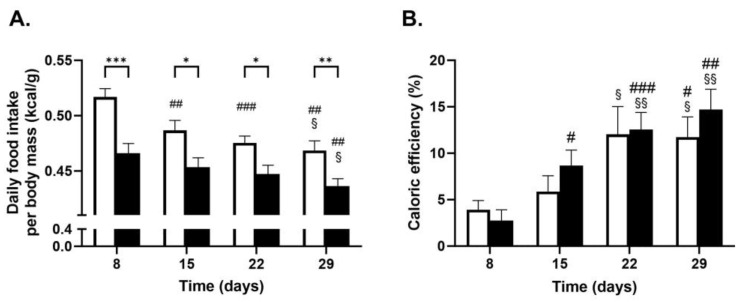
Effect of GCE treatment on daily food intake per body mass and feed efficiency. Daily food intake and body mass were measured at the indicated days in CTRL (CTRL, ☐, and *n* = 10) and GCE (GCE, ■, and *n* = 10) groups treated as described in Figure 1. Results are the means ± SEM of the ratio of daily food intake (kcal) per body mass (**A**) and feed efficiency (body weight gain from Day 1 per daily caloric intake). (**B**) Statistical analyses were performed using 2-way ANOVA (* *p* < 0.05, ** *p* < 0.01, or *** *p* < 0.001 comparing CTRL and GCE groups; # *p* < 0.05, ## *p* < 0.01, and ### *p* < 0.001, compared to Day 8 of the same group; and § *p* < 0.05 and §§ *p* < 0.01, compared to Day 15 of the same group).

Interestingly, analysis revealed small but significantly lower ratios of daily caloric intake per body mass in the GCE group as compared to CTRL on Days 8, 22, and 29 (Figure 2). This parameter significantly decreased with time in the CTRL group but was not significantly different in the GCE group, except for Day 29. However, kcal, or caloric efficiency, which is calculated as body weight gain divided by caloric intake × 100, was not significantly different between the CTRL and GCE groups for all time points. Efficiency values increased significantly in the GCE group at all time points when compared to Day 8, while those of the CTRL group were significantly increased only on Day 29 when compared to Day 8, or on Days 22 and 29 when compared to Day 15. Similar results were observed for food efficiency, which is calculated using food intake as grams instead of kcal.

#### 2.1.2. Plasma Lipid, Lipoprotein, and Glucose Measurements

**Figure 3:**
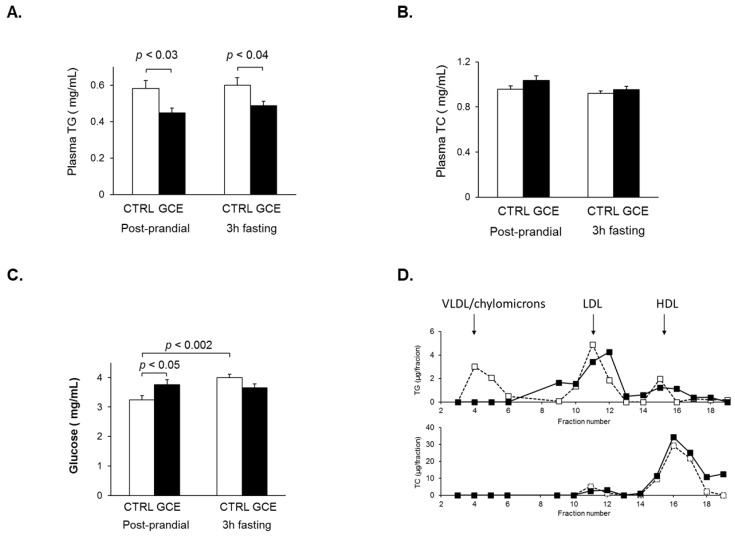
Effect of GCE on plasma lipid and glucose levels and lipoprotein profiles in C57BL/6J mice. C57BL/6J mice were treated with GCE as described in Figure 1 (GCE, *n* = 10, and ■; CTRL, *n* = 10, and ☐). On day 30 of treatment, blood samples from CTRL and GCE groups were taken between 8:00 AM and 9:00 AM, representing the post-prandial period, and after a 3 h fasting period (3 h fasting), from which plasma was isolated and plasma triglycerides (TG) (**A**), total cholesterol (TC) (**B**), and glucose (**C**) levels were determined. Results are shown as means ± SEM; all *p*-values were calculated from Student’s *t*-test. (**D**) On day 40 of treatment, mice were anesthetized, and blood samples were obtained via cardiac puncture. Plasma was isolated and pooled from 3 mice from each group, from which a lipoprotein profile was obtained using gel filtration chromatography as described in Materials and Methods. Fractions were analyzed for TG (top panel) and TC (bottom panel) content. The elution of TG-rich lipoproteins, including VLDL and chylomicrons, LDL, and HDL, is indicated with arrows.

Plasma TG levels were measured both at the end of the dark cycle (between 8:00 and 9:00 AM), considered as the post-prandial period, as well as at noon after a 3 h fasting period [20]. For both time points, plasma TG levels were significantly lower in the GCE group (Figure 3A), consistent with previous reports showing a hypotriglyceridemic effect of GCE [5,24,25]. No significant difference was observed in plasma TC between the two groups (Figure 3B). Furthermore, analysis of lipoprotein profiles obtained from a pool of plasma from three animals from each group revealed the absence of a peak corresponding to TG-rich lipoproteins (VLDL and chylomicrons) in GCE-treated mice (Figure 3D), which would be consistent with the observed lower levels of plasma TG. Plasma glucose levels were relatively high in both groups and significantly different between the GCE and CTRL groups, but only in plasma obtained at 9:00 AM (Figure 3C). However, plasma insulin (CTRL 0.34 ± 0.019 ng/mL; GCE 0.36 ± 0.020 ng/mL) was not significantly different between the two groups, suggesting an absence of glucose intolerance or insulin resistance. Plasma leptin, which also participates in regulating peripheral insulin sensitivity, was not found to be significantly different in either group (CTRL 0.27 ± 0.059 ng/mL; GCE 0.36 ± 0.058 ng/mL) [26].

#### 2.1.3. Analysis of Tissue Lipid Content

Lipid composition analysis of tissues revealed a statistically significant increase in total TC and TG levels in livers from the GCE group as compared to those from CTRL (Table 2). Phospholipid (PL) content remained unchanged between the two groups, so this increase remained significant if hepatic TC content was expressed relative to PL content (Table 2).

In adipose tissue, there was no significant difference in TG levels. However, small but statistically significantly higher levels in the adipose tissue TG/PL ratio were observed for the GCE as compared to the CTRL groups. Interestingly, analysis revealed a 30% increase in skeletal muscle TG content in GCE animals as compared to CTRL mice. No significant differences were observed when comparing the levels of heart TG or TC content between the two groups.

#### 2.1.4. Changes in Hepatic Protein Levels in GCE-Treated Mice

**Figure 4:**
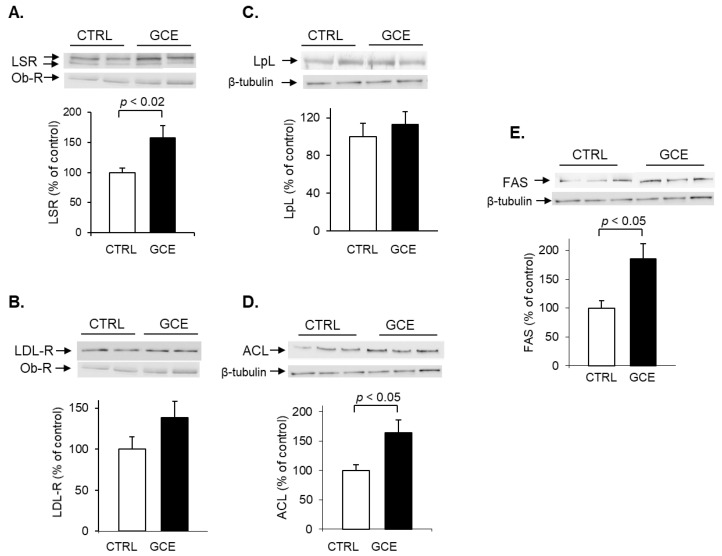
Effect of GCE on hepatic protein levels of lipoprotein receptors and enzymes involved in TG metabolism. C57BL/6J mice were treated with GCE as described in Figure 1. On day 40 of treatment, mice were sacrificed, and livers were excised. Liver membranes and non-membrane fractions were prepared. Proteins from each fraction were separated on SDS-PAGE, followed by immunoblots to detect (**A**) LSR and (**B**) LDL-R in the total membrane fractions, (**C**) lipoprotein lipase (LpL), (**D**) ACL, and (**E**) FAS in the non-membrane fractions prepared from livers of CTRL (☐) and GCE (■) groups. For LSR and LDL-R, the leptin receptor (Ob-R) was used as internal control as its protein levels did not significantly change between each group of animals. For LpL, ACL, and FAS, β-tubulin was used as the internal control. Representative immunoblots are shown (top panels), as well as results of densitometric analyses (bottom panels) as means ± SEM (*n* = 5–8 per group); *p*-values are shown in the figure and were calculated using Student’s *t*-test.

Immunoblots of solubilized protein from total liver membranes were performed to detect the lipoprotein receptors, LSR and LDL-R, which are lipoprotein receptors. Results revealed a 57% increase (Figure 4A, *p* < 0.02) in LSR protein levels of the GCE group in mouse hepatic liver membrane fraction as compared to that of the CTRL group. LDL-R protein levels were not statistically significantly different between the CTRL and GCE groups (Figure 4B).

Internalization of TG-rich lipoproteins with LSR requires the presence of fatty acids to activate LSR. Fatty acids are lipolytic products produced following the hydrolysis of lipoprotein TG via lipoprotein lipase (LPL), which is bound to the endothelial cell walls in the space of Disse [27,28]. Immunoblots were therefore also performed to detect hepatic LpL; protein levels were not found to be different in the GCE group as compared to CTRL (Figure 4C).

Immunoblots of liver non-membrane fractions were performed to detect hepatic ACL, which was surprisingly found to be higher in the GCE group as compared to the CTRL group (Figure 4D) after 40 days of treatment. Moreover, fatty acid synthase (FAS), which acts downstream of ACL, was also found to be significantly increased in GCE-treated animals (Figure 4E).

**Figure 5:**
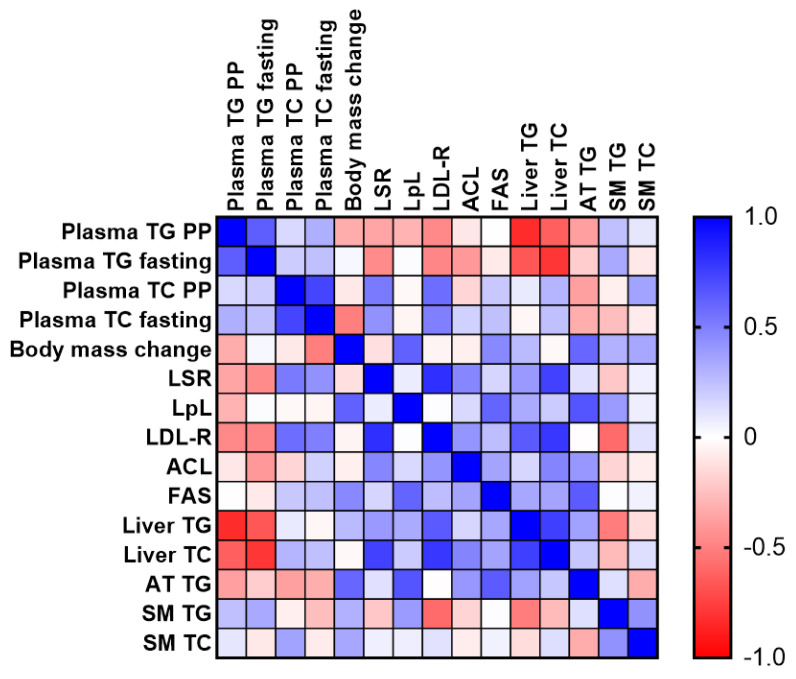
Correlation matrix comparing plasma and tissue lipid parameters with liver proteins from both CTRL and GCE groups. Correlations between different parameters are shown as heatmap, ranging from +1.0 (blue) to −1.0 (red) (PP, post-prandial; AT, adipose tissue; and SM, skeletal muscle).

Correlational analysis was then performed on the combined data of the CTRL and GCE groups. A heat matrix plot revealed that liver TC and TG were strongly and inversely correlated with both plasma and post-prandial TG levels; this was not the case for adipose tissue or muscle lipid levels (Figure 5, Table S1). Liver TC was strongly correlated with both LSR and LDL-R, while liver TG was correlated with only LDL-R. Correlational analysis also revealed a strong association between hepatic LSR and LDL-R protein levels.

### 2.2. Treatment of Hepa1-6 Cells with HCA

**Figure 6:**
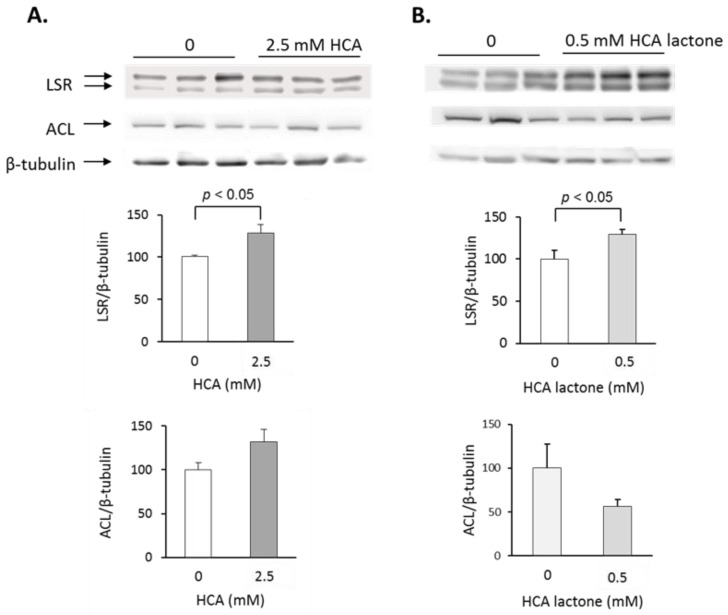
Effect of HCA or the lactone form of HCA (HCA-lactone) on LSR and ACL protein levels in Hepa1-6 cells. Hepa1-6 cells at 80–90% confluency were incubated with the indicated concentrations of (**A**) HCA or (**B**) HCA-lactone for 18 h at 37 °C, followed by cell lysis to extract total proteins. Immunoblots were performed on the cell protein extracts to detect LSR or ACL, as described in Methods and Materials. Densitometric analyses were performed using β-tubulin as the loading control. Results are shown as means ± SEM (*n* = 3). Statistical analyses were performed using Student’s *t*-test; *p* values are shown in the figure.

We questioned if the observed increase in LSR protein levels was due to the HCA, which is present in GCE as a potassium salt or as a lactone [29]. To test this, mouse Hepa1-6 cells [21] were incubated for 18 h at 37 °C with HCA in either form. Immunoblots revealed that treatment with 2.5 mM HCA as a potassium salt (Figure 6A) or 0.5 mM of HCA lactone (Figure 6B) led to increased LSR protein levels. No statistically significant changes in Hepa1-6 ACL protein levels were observed in the presence of either form of HCA.

## 3. Discussion

The results from the in vivo study show that GCE treatment lowered TG levels in plasma obtained during the post-prandial state and after a 3 h fasting period. Hepatic LSR protein levels were increased, which would be consistent with the observed decrease in plasma TG-rich lipoproteins in GCE-treated mice (Figure 3D). For this study, we focused on LSR, since it has been shown to play an important role in the removal of these lipoproteins during the post-prandial period [20]. Reduced LSR leads to moderate hypercholesterolemia and hypertriglyceridemia in *lsr*+/− mice [20,30]. Furthermore, adenovirus-mediated expression of LSR normalizes plasma TG levels in hypertriglyceridemic, obese mouse models that are LSR-deficient [30]. LDL-R plays a supportive role in the removal of TGRL [31]. However, protein levels of this receptor were not statistically significantly increased in the GCE group as compared to those of the CTRL group. The observed GCE-induced LSR increase supports our hypothesis that GCE treatment can lead to an increase in receptors that mediate the hepatic uptake of TG.

Cell culture experiments confirmed the in vivo results, showing that 18 h treatment of Hepa1-6 cells with the GCE active ingredient, HCA, led to increased LSR protein levels. A previous study reported increased HMG CoA reductase and LDL-R activities in human HepG2 cells treated with HCA under similar conditions, postulating that this may be due to HCA inhibition of lipogenesis, thus blocking the production of acetyl CoA, which serves as a precursor for cholesterol synthesis [32]. The SREBP pathway acting as a cholesterol sensor would then increase transcription of HMG CoA reductase and LDL-R. Here, a strong association was observed between liver total cholesterol levels and not only LDL-R but also LSR, as well as between hepatic LDL-R and LSR, which could indicate potential common regulatory pathways for these two receptors. Since the SREBP gene was downregulated in *lsr*+/− mice who display moderate hypertriglyceridemia [22], we would propose SREBP-dependent transcription as a potential mechanism for HCA-induced increases in LSR, similar to what was observed for LDL-R [32]. Additional investigation is required to confirm this mechanism of action.

The correlation between LDL-R and LSR also supports the notion of positive cooperativity between these two lipoprotein receptors. Indeed, elevated post-prandial lipemia in *lsr*+/− mice was increased further when these mice were crossed with LDL-R−/− mice [20]. Although the LDL-R is the principal receptor for LDL and LSR has the highest affinity for TG-rich lipoproteins, only liver TC was significantly correlated with both LDL-R and LSR, while liver TG content was found to be significantly correlated only with LDL-R. We speculate that this absence of correlation may be due to the cumulative GCE effect on TG production and TG uptake, but additional investigation is required to determine the underlying mechanisms.

Significant increases in total liver TG and TC were observed after 40-day GCE treatment. A strong inverse correlation was observed with plasma TG, which would be consistent with increased hepatic TGRL uptake but could also be a result of increased TG production. Indeed, hepatic protein levels of ACL were actually increased in the GCE group, which may be a result of the extended period (40 days) of repressed fatty acid synthesis using the active HCA ingredient in GCE [33]. We would speculate that the increased influx of lipids following lipoprotein uptake could contribute to increased production of acetyl-CoA and citrate, requiring an increase in ACL, thus overriding inhibition with HCA. Indeed, the GCE treatment has been shown to be only partially effective in decreasing liver lipid levels to baseline in cases of a high influx of lipids that occurs following a high-fat diet [34,35,36]. Therefore, liver TG levels represent both those acquired following increased influx from the circulation as well as those that were endogenously produced.

Here, animals were on a standard chow diet, and there was no significant difference in weight gain in this study, most likely because weight loss in other studies was based on obesity or diet-induced obesity mouse models [37,38,39], while here, dietary lipid intake was only moderately elevated (Table 1, 23–24% kcal) due to the corn oil vehicle used for oral administration of GCE. Also, young adult mice were used, so any weight gain is most likely due to age, maintenance, and the increased fat content of the corn oil in the vehicle rather than from growth. GCE has been reported to suppress appetite, but the results have been variable in both animal and clinical data [39]. Here, no significant differences in absolute food intake were observed between CTRL and GCE groups after the first week, and the amount of kcal consumed per day was similar to those in the literature [40]. Caloric, or food efficiency, is a measure of the degree to which food is digested, absorbed, and utilized by the organism and was not found to be different in GCE-treated mice as compared to CTRL animals. However, the daily food intake relative to body mass gain was significantly lower in the GCE-treated animals as compared to CTRL mice on a standard chow diet. Other studies have reported either no change or decreased food efficiency following GCE supplementation [41], but in these reports, diets with high caloric density were used [42,43,44]. Analysis showed that changes in body mass and adipose tissue TG content were correlated in this study, which would be consistent with previous studies reporting loss of body mass and decreased adipose tissue following GCE treatment [24]. It would therefore seem that the GCE may affect metabolism rather than changes in food efficiency.

Interestingly, skeletal muscle TG levels were increased in the GCE group, which could be indicative of hyperglycemia and insulin resistance [45,46]. However, while glucose levels were higher in GCE mice, no difference in fasting insulin or leptin levels was observed in this study. A previous study did, however, report increased β-oxidation in the muscle of HCA-treated mice [47], which has been suggested to be a compensatory response to lipid accumulation in order to avoid the development of insulin resistance [48].

These data confirm our hypothesis that increased receptor-mediated uptake of TGRL by increasing hepatic lipoprotein receptors can contribute to the hypotriglyceridemic effect of GCE. We speculate that this increased lipid influx may actually override HCA inhibition of ACL, which may explain the observed accumulation of liver lipids. The limitations of the current study require additional mechanistic studies to confirm this. Nevertheless, we can conclude that while the use of GCE could be beneficial with regard to a reduction in weight gain and plasma TG levels [6], chronic use could potentially increase the risk of hepatic and skeletal muscle lipid accumulation, leading to adverse effects on liver lipid homeostasis and function [49,50,51,52,53,54], and potential complications including insulin resistance.

## 4. Materials and Methods

### 4.1. Materials

Reagents were purchased from Sigma-Aldrich (St. Quentin Fallavier, France), unless otherwise specified. GCE used was in the form of Pure Super Citri Max (Chatsworth, CA, USA). Anti-LSR (HPA007270) and anti-β-tubulin (T5201) antibodies were obtained from Sigma-Aldrich, anti-LDL-R antibodies (ab30532) from Abcam (Paris, France), and anti-FAS antibodies from BD Biosciences (610962, Le Pont de Claix, France). Anti-ACL antibodies (4332S) and secondary anti-rabbit (7074S) and anti-mouse (7076S) peroxide-conjugated IgG were purchased from Cell Signaling Technology (Boston, MA, USA). Anti-LpL (sc-373759) and rabbit peroxidase-conjugated anti-goat IgG (sc-2922) antibodies were obtained from Santa Cruz Biotechnology (Heidelberg, Germany).

### 4.2. Cell Culture Studies

The mouse hepatoma cell line, Hepa1–6 (DSMZ, Brunswick, Germany), was maintained in Dulbecco’s Modified Eagle Medium containing 10% heat-inactivated fetal bovine serum and 1 mM glutamine [21]. Cells were seeded in 12-well plates and used after 48 h at 80–90% confluence. On the day of the experiment, cells were washed in phosphate-buffered saline (PBS) and then incubated at 37 °C in a 95% air, 5% CO_2_ environment for 18 h with HCA in its potassium salt or lactone form in 1% (*v*/*v*) DMSO; control cells were treated with vehicle (1% (*v*/*v*) DMSO) alone. After incubation, cell lysates were prepared using ice-cold RIPA buffer containing anti-proteases [55]. Protein was measured using the BCA assay (Thermo Scientific, Courtaboeuf, France).

### 4.3. Animals and Diets

C57BL/6J female mice (10-weeks old, Janvier Breeding, Le Genest Saint Isle, France) were housed in certified animal facilities in a 12 h light–dark cycle (the light cycle was between 8:00 AM and 8:00 PM), maintained at 21–22 °C with a humidity of 50 ± 20%, and given ad libitum access to a standard chow diet (Teklad Global 18% Protein Rodent Diet 2018, Envigo, Gannat, France) and water. This is a grain-based diet, with soybean oil providing the source of fat (Table 1). Animal handling and experimental protocols were authorized by the institutional review board in accordance with the European Communities Council Directive of 24 November 1986 (86/609/EEC) for the use and care of laboratory animals and in conformity with PHS policy on Humane Care and Use of Laboratory Animals, incorporated in the Institute for Laboratory Animal Research (ILAR) Guide for Care and Use of Laboratory Animals (authorization n° 54-547-24).

### 4.4. GCE Treatment

GCE was obtained from Pure Super Citri Max capsules (Chatsworth, CA, USA) containing 80% extract, of which 60% represents hydroxycitrate; the remaining ingredients included 10% calcium, gelatin, water, and magnesium stearate. GCE was added to a solution of 50% (*v*/*v*) corn oil in physiological saline for a final concentration of 20 mg GCE in 300 µL. This dose was determined in a pilot study that induced a lower weight gain as compared to controls. It corresponds to a dose of 1 g GCE/kg body weight (equivalent to 480 mg or 2.3 mmol HCA/kg/day), similar to those used in previous rodent studies [37,56,57].

Animals from the same lot were separated into two groups. One group received the 50% corn oil preparation containing GCE (GCE group), and the control (CTRL) group received the vehicle alone. All animals received the vehicle alone (CTRL) or the vehicle containing GCE (GCE) daily via oral administration (gavage of 300 µL/day) at 6:00 PM before the dark active cycle (*n* = 10 animals per group). All animals received the same standard chow diet that was provided ad libitum. Body weight was monitored twice per week, and food intake was measured weekly (days 8, 15, 22, and 29). Caloric efficiency was calculated as the body weight gain (from Day 1) divided by the daily dietary intake as kcal × 100.

Blood samples were taken from the retro-orbital cavity of mice lightly anesthetized with isoflurane using capillary tubes coated with 5% EDTA. Sampling was performed on day 0 and day 30 of the study at 9:00 AM and at noon after a 3 h fasting period. Blood was immediately centrifuged at 13,000× *g* at 4 °C for 5 min, and plasma was removed and stored at −20 °C for analysis. On day 40, mice were euthanized (anesthesia overdose using isoflurane gas). Liver, heart, skeletal gastrocnemius muscle, and epididymal fat pads were removed, rinsed in PBS, snap-frozen in liquid N2, and stored at −80 °C for later analysis.

### 4.5. Lipid and Glucose Analyses of Plasma and Tissues

Lipid and glucose levels in plasma and lyophilized tissue lipid extracts were measured using enzymatic colorimetric kits (for TG, total cholesterol (TC), and glucose, Biomérieux, Craponne, France; for phospholipid (PL), Sobioda, Montbonnot, France) as described previously [20]. Plasma leptin (R & D, Lille, France) and insulin (Millipore, Billerica, MA, USA) levels were measured in the samples obtained after the 3 h fasting period using ELISA assays, according to the manufacturer’s instructions.

### 4.6. Lipoprotein Profiles

Equal volumes of plasma samples obtained at noon from 3 mice were pooled and applied to a Superose 6 10–300 GL (GE Healthcare, Orsay, France) on an AKTA Explorer 10 in 30 mM phosphate buffer containing 150 mM of NaCl, 1 mM of EDTA, and 0.02% NaN3, pH 7.4, at 0.2 mL/min [20]. Fractions (500 µL) were collected and analyzed for TC and TG content as described above.

### 4.7. Immunoblotting Studies

Immunoblots were performed on proteins solubilized from cell lysates, liver total membranes, or cytosolic fractions prepared as previously described [21]. Primary antibody dilutions were 1:1000 except for those from Santa Cruz Biotechnology, which were used at a dilution of 1:500. Protein content was determined using the modified Lowry assay [58]. Identical amounts of protein were applied to each gel lane. Normalization was performed using bands revealed by the detection of endogenous controls: anti-Ob-R (sc-8391, Santa Cruz) for liver total membranes and anti-β-tubulin for cell lysates or liver cytosolic fractions. Protein bands were detected using HRP-linked secondary antibodies and a chemiluminescence kit (GE Healthcare) and revealed using the Bio-Rad Fusion FX5 (Vilber Lourmat, France). Image analysis was performed using the freeware ImageJ, v1.54g.

### 4.8. Statistical Analyses

The sample size of the animal study was determined with a power analysis [59]. The results are represented as means ± SEM, unless otherwise indicated. The parametric Student’s *t*-test or a 2-way ANOVA was used to determine statistical significance between groups (as indicated in each figure), with statistical significance considered as *p* < 0.05.

## Figures and Tables

**Table 1:** Food intake of mice treated with GCE ^1^.

	Food Intake (kcal/Day)		Fat Intake (% kcal/Day)	
Day	CTRL	GCE	CTRL	GCE
8	12.8 ± 0.14	11.9 ± 0.22 **	23.1 ± 0.09	23.7 ± 0.16 **
15	12.3 ± 0.15	11.9 ± 0.22	23.4 ± 0.10	23.8 ± 0.16
22	12.3 ± 0.21	11.8 ± 0.21	23.4 ± 0.14	23.8 ± 0.15
29	12.1 ± 0.23 #	11.6 ± 0.18	23.6 ± 0.16 #	23.9 ± 0.13

^1^ Ten-week-old female C57BL/6J mice received 300 µL of 50% corn oil in physiological saline alone (CTRL, *n* = 10) or containing 20 mg of GCE (GCE, *n* = 9) per day for 40 days, during which they received a standard chow diet (Table 1) and water ad libitum. Food intake was monitored on the indicated days, and the total daily kcal intake was calculated to include the amount of corn oil consumed via gavage. Fat intake is indicated as % of total kcal consumed per day. Results are shown as means ± SEM (** *p* < 0.01, GCE compared to CTRL, and # *p* < 0.05, versus Day 8 CTRL, using 2-way ANOVA).

**Table 2:** Tissue lipid content following 40-day treatment of GCE ^1^.

	Liver Total Liver Content (mg)		Adipose Tissue µg/mg Dry Weight		Skeletal Muscle µg/mg Dry Weight		Heart µg/mg Dry Weight	
Lipid	CTRL	GCE	CTRL	GCE	CTRL	GCE	CTRL	GCE
TC	1.4 ± 0.1	2.0 ± 0.1 **	nd	nd	2.5 ± 0.3	3.0 ± 0.2	5.9 ± 0.5	5.8 ± 0.5
TG	7.8 ± 0.7	9.6 ± 0.5 *	9.6 ± 1.1	9.7 ± 0.61	11.5 ± 0.7	15.0 ± 1.1 *	19.3 ± 2.5	17.9 ± 2.6
PL	8.5 ± 0.5	8.3 ± 0.3	3.5 ± 0.4	3.1 ± 0.24	20.6 ± 2.0	24.4 ± 1.6	24.2 ± 3.0	25.6 ± 3.8
Ratio								
TC/PL	0.17 ± 0.03	0.25 ± 0.02 *	--	--	0.12 ± 0.01	0.12 ± 0.01	0.25 ± 0.03	0.25 ± 0.1
TG/PL	0.92 ± 0.1	1.18 ± 0.1	2.77 ± 0.1	3.14 ± 0.14 *	0.53 ± 0.01	0.63 ± 0.04	0.83 ± 0.2	0.63 ± 0.2

^1^ Total cholesterol (TC), triglycerides (TG), and phospholipid (PL) contents in different murine tissues (control group, CTRL—*n* = 10; GCE-treated group, GCE—*n* = 10). Adipose tissue samples were from epididymal fat pads, and those of skeletal muscle were the gastrocnemius muscle. Absolute values are expressed as total liver content or as µg/mg dry weight, as indicated. Ratios relative to PL content are shown for each tissue and each group. Results are shown as means ± SEM. (* *p* < 0.05 and ** *p* < 0.01, GCE compared to CTRL using Student’s *t*-test; nd: not determined).

## Data Availability

All data are contained within the article.

## Supplementary Materials

The supporting information can be downloaded at https://www.mdpi.com/article/10.3390/ijms242216298/s1.
