# Supplementary material for: Garcinia cambogia Extract Increased Hepatic Levels of Lipolysis-Stimulated Lipoprotein Receptor and Lipids in Mice on Normal Diet
Source: Int J Mol Sci. 2023 Nov 14;24(22):16298. doi: 10.3390/ijms242216298 (PMC10671705; doi:10.3390/ijms242216298)
Supplement: Supplementary file 1 [file ijms-24-16298-s001.zip › ijms-2684924-supplementary.pdf]

Table S1. *Correlations of plasma and tissue lipid parameters with liver proteins from both the CTRL and GCE groups of mice*[1]

	Plasma TG PP	Plasma TG Fasting	Plasma TC PP	Plasma TC Fasting	Body mass change	LSR	LpL	LDL-R	ACL	FAS	Liver TG	Liver TC	AT TG	SM TG	SM TC
**Plasma TG PP**	1.00	**0.63**	0.15	0.31	-0.33	-0.36	-0.30	-0.47	-0.10	-0.01	**-0.83**	**-0.63**	-0.38	0.24	0.09
**Plasma TG fasting**		1.00	0.20	0.25	0.03	-0.46	0.01	-0.48	-0.41	-0.09	**-0.67**	**-0.79**	-0.20	0.34	-0.09
**Plasma TC PP**			1.00	**0.73**	-0.09	0.52	-0.03	**0.57**	-0.17	0.21	0.08	0.29	-0.38	-0.07	0.36
**Plasma TC fasting**				1.00	-0.51	0.43	-0.04	0.50	0.18	0.24	-0.03	0.25	-0.32	-0.26	-0.08
**Body mass change**					1.00	-0.13	**0.62**	-0.05	-0.07	0.46	0.27	-0.03	**0.60**	0.30	0.34
**LSR**						1.00	0.07	**0.81**	0.47	0.16	0.40	**0.74**	0.12	-0.22	0.06
**LpL**							1.00	<0.01	0.15	**0.61**	0.33	0.20	**0.6**	0.39	0.06
**LDL-R**								1.00	0.42	0.26	**0.64**	**0.78**	-0.01	-0.58	0.11
**ACL**									1.00	0.35	0.16	0.48	0.41	-0.17	-0.07
**FAS**										1.00	0.35	0.36	**0.64**	<0.01	0.05
**TG**											1.00	**0.75**	0.37	-0.51	-0.13
**TC**												1.00	0.22	-0.27	0.13
**AT TG**													1.00	0.12	-0.33
**SM TG**														1.00	0.42
**SM TC**															1.00

[1] Pearson's correlation coefficient; statistically significant correlations ($p<0.05$) are shown in bold (PP = postrpandial).
